# Interactions of polymorphisms in different clock genes associated with circadian phenotypes in humans

**DOI:** 10.1590/S1415-47572010005000092

**Published:** 2010-12-01

**Authors:** Mario Pedrazzoli, Rodrigo Secolin, Luiz Otávio Bastos Esteves, Danyella Silva Pereira, Bruna Del Vechio Koike, Fernando Mazzili Louzada, Iscia Lopes-Cendes, Sergio Tufik

**Affiliations:** 1 Escola de Artes, Ciências e Humanidades, Universidade de São Paulo, São Paulo, SPBrazil; 2 Departamento de Genética Médica, Universidade Estadual de Campinas, Campinas, SPBrazil; 3 Departamento de Psicobiologia, Universidade Federal de São Paulo, São Paulo, SPBrazil; 4 Departamento de Fisiologia, Universidade Federal do Paraná, Curitiba, PRBrazil

**Keywords:** clock genes, gene interaction, morningness-eveningness, sleep, circadian rhythm

## Abstract

Several studies have shown that mutations and polymorphisms in clock genes are associated with abnormal circadian parameters in humans and also with more subtle non-pathological phenotypes like chronotypes. However, there have been conflicting results, and none of these studies analyzed the combined effects of more than one clock gene. Up to date, association studies in humans have focused on the analysis of only one clock gene per study. Since these genes encode proteins that physically interact with each other, combinations of polymorphisms in different clock genes could have a synergistic or an inhibitory effect upon circadian phenotypes. In the present study, we analyzed the combined effects of four polymorphisms in four clock genes (*Per2*, *Per3*, *Clock* and *Bmal1*) in people with extreme diurnal preferences (morning or evening). We found that a specific combination of polymorphisms in these genes is more frequent in people who have a morning preference for activity and there is a different combination in individuals with an evening preference for activity. Taken together, these results show that it is possible to detect clock gene interactions associated with human circadian phenotypes and bring an innovative idea of building a clock gene variation map that may be applied to human circadian biology.

## Introduction

In the last two decades, there has been significant progress in understanding the molecular basis of mammalian circadian rhythmicity (Ko and Takahashi, 2006, Dardente and Cermakian, 2007). Mutations in clock genes are associated with abnormal circadian parameters, including sleep in animals (Ralph and Menaker, 1988; Toh *et al.*, 2001; Iwase *et al.*, 2002; Taheri and Mignot, 2002).

Very similar circadian phenotypes to those observed in animals may be recognized in humans. The first report regarding the influence of a clock gene associated with a circadian rhythm phenotype in humans appeared in 1998. In that study, Katzenberg *et al.* (1998) reported that a polymorphism in the *Clock* gene was associated with diurnal preferences. This result was replicated in a Japanese sample (Mishima *et al.*, 2005), but not in a British population sample or in a Brazilian sample (Robiliard *et al.*, 2002; Pedrazzoli *et al.*, 2007).

Polymorphisms in other clock genes were also reported to be associated with circadian phenotypes. Ebisawa *et al.* (2001) found that polymorphisms in the *Per3* gene could be associated with Delayed Sleep Phase Syndrome (DSPS). Archer *et al.* (2003) reduced the region of influence of the *Per3* gene in DSPS to a variable number of tandem repeats (VNTR) (four or five repeats) localized in exon 18, and reported that the shorter allele was associated with DSPS. Pereira *et al.* (2005) also reported that this VNTR in the *Per3* gene was associated with DSPS, but curiously they found that the longer allele was associated with the disease. As the two studies were performed in populations with a Caucasian European genetic background, living at very different latitudes, these conflicting results suggest that latitude can affect the expression of clock genes. Archer *et al.* (2003) and Pereira *et al.* (2005) also found that the VNTR polymorphism in the *Per3* gene is associated with morning-evening tendencies. Viola *et al.* (2006) have studied the effects of the *Per3* VNTR polymorphism in regulation of diurnal preferences more extensively, under laboratory controlled conditions, and have found that it has predictive value in the response to sleep loss.

Polymorphisms in the *Per1* and *Per2* genes have also already been associated with morning-evening tendencies in a British population sample (Carpen *et al.*, 2005; Carpen *et al.*, 2006). Additionally, a mutated *Per2* allele was shown to be associated with a familial case of Advanced Sleep Phase Syndrome (ASPS) (Toh *et al.*, 2001), but these data were not replicated in a family with ASPS in Japan (Satoh *et al.*, 2003).

As shown above, these previous studies, that have been carried out in several populations around the world, show that polymorphisms in clock genes are independently associated with circadian phenotypes. Nevertheless, they have produced conflicting results, and none of these studies have analyzed the combined effects of two or more genes. The knowledge accumulated up to date shows that the proteins coded by different clock genes interact physically with each other and act as are transcription factors; therefore, it is possible to speculate that combinations of polymorphisms in these genes may influence phenotype, and that a combined analysis of the effects of different clock genes may be more accurate and more informative than single gene analyses.

In the present study, we analyzed the possibility of detecting interactions between polymorphisms in different clock genes associated with circadian phenotypes in humans.

## Materials and Methods

###  Subjects

####  Volunteers 

From an initial screening of 1500 volunteers, a total of 98 volunteers of extreme types were selected to participate based on the Horne-Östberg (HO) questionnaire score (1976). A group composed of extreme morning subjects (n = 47) with a mean HO score of 68.1 ± 4.5, 82% Caucasian, 71.3% female, mean age 21.9 ± 2.7 was compared with a group of extreme evening subjects (n = 51), mean HO score of 28.8 ± 5.6, 80% Caucasian, 71.6% female, mean age 22.9 ± 3.3. The study was approved by the Committee on Ethics of the Universidade Federal de São Paulo (# 0790/02, # 0617/02, and # 1453/06). Volunteers were undergraduate students living in their own places, and all were synchronized to have activities during the day and nocturnal rest, attending morning (8:00-12:00) and/or afternoon (14:00-18:00) classes from Monday to Friday. The participants signed an informed consent to participate.

###  Genotyping clock genes polymorphisms

Blood samples were taken from all participants and DNA was extracted from white blood cells (Miller *et al.*, 1988). Four polymorphisms of four clock genes, *Clock*, *Per3*, *Per2 and Bmal1*, were typed as follows:

For the *Clock* gene polymorphism T3111C (AF11568), PCR-RFLP was done as described by Pedrazzoli *et al.* (2007). For the *Per3* gene VNTR polymorphism (four or five repeats), PCR was done as described by Ebisawa *et al.* (2001). For the *Per2* C311G polymorphism (rs2304672) an assay-on-demand (assay ID:C2129919_1; Applied Biosystems, Foster City, CA, USA) was used as described by Carpen *et al.* (2005), and finally we used the DHPLC system using a semi-denaturing temperature of 59.7 °C (Wave system, Transgenomic, Inc. USA) for the *Bmal1* promoter region A-1420G polymorphism (rs4757138), which is the only polymorphism in this study not previously reported to be associated with any phenotype. The PCR fragment for DHPLC analysis was obtained using the primers F 5'ggcagagacaaaggagcaat 3' and R 5'cagcgtcttctttttcg 3', designed using the TFSEARCH program ver.1.3 and included the RORα transcription factor binding site.

###  Statistical Analysis

Initially, all polymorphisms were evaluated with regard to the Hardy-Weinberg equilibrium using ARLEQUIN v. 3.1 software (Excoffier *et al.*, 2005). We designed and implemented an algorithm in PERL script (*combination.pl*) to generate all possible combinations among the polymorphisms, and also calculated the frequencies of these combinations within the two experimental groups.

In order to test whether differences in combination frequencies between groups were statistically significant, we performed a *log* ratio test (LRT) using EXACT TEST v. 1.0.0.2 software (EDNData). To avoid bias due to previous gene associations within combinations, we separately performed Fisher's exact test for each gene using the *fisher.test* function in the R environment software (R Development Core Team 2007). In addition, combinations of two, three and four SNPs were analyzed. Multiple comparisons in all these statistical tests were corrected using a False Discovery Rate (FDR) correction and the *p.adjust* function in R environment (Storey and Tibshirani, 2003; R Development Core Team 2007). Corrected p-values (p_corr_) < 0.05 were considered to be statistically significant (Storey and Tibshirani, 2003).

## Results

**Figure 1:**
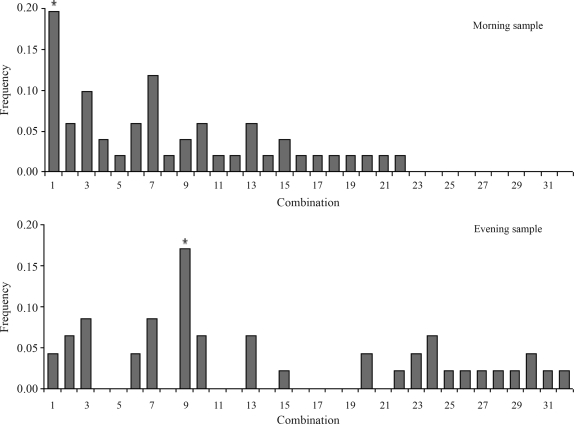
Frequency distribution of the polymorphism combinations in the morning and evening samples, when considering combinations of four polymorphisms. Significant difference between groups, *p < 0.05.

No associations were observed when each polymorphism was considered individually (*Clock* T311C p_corr_ = 0.7839; *Per3* p_corr_ = 0.4113; *Per2* p_corr_ = 1.0000; *Bmal1* p_corr_ = 0.4113), or when combinations of two or three polymorphisms were tested. Nevertheless, when analyzing the combination of four polymorphisms it became evident that there were statistically significant differences in the frequency distribution (p_corr_ = 0.0330) between combination #1 (morning sample) and combination #9 (evening sample) (Figure1). We found 31 different four polymorphism combinations in our sample out of the 81 that are mathematically possible, obviously the combinations involving low frequency alleles are much less likely to be found (Table 1).

Interestingly, combinations #1 #9 only differed in their *Bmal1* genotypes, which are homozygous G and heterozygous A/G, respectively. They shared the same genotypes for the other clock genes analyzed, *i.e.*, they were heterozygous for the *Per3* VNTR, homozygous C for the *Per2* SNP and homozygous T for the *Clock* SNP*.*

## Discussion

This study was designed to evaluate the possibility of observing the combined effects of polymorphisms in four different clock genes, two in the positive arm (*Clock* and *Bmal1*) and the other two in the negative arm (*Per3 and Per2*) of the main molecular loop of circadian rhythms.

Statistical analysis demonstrated that there could be an interaction among the gene polymorphisms, which would give a tendency to determined chronotypes. This hypothesis is supported by the fact that the only difference between combinations #1 and #9 is the *Bmal1* polymorphism, but this polymorphism alone did not provide any evidence of association. In fact, it seems that specific combinations of polymorphisms among different clock genes are stronger markers to chronotypes than single polymorphisms.

The cellular clock function hypothesis proposes that the molecular mechanism responsible for clock function operates as a negative feedback loop in which the clock encoded proteins physically interact with each other, whereby *Clock* and *Bmal1* also act as transcription factors within and outside of the loop (Lowrey *et al.*, 2000). It is therefore reasonable to hypothesize that subtle changes in protein structure, due to non-synonymous polymorphisms, could alter the dimerization rates of proteins, thus causing subtle alterations in the circadian regulation and leading to slightly different phenotypes, as seen with the different chronotypes. In addition, subtle changes in the promoter or regulatory regions could affect rates of gene expression, thus changing the phase relationship between clock components and leading to subtle differential regulation of circadian rhythms.

At this stage of the research it is still difficult to propose a mechanism by which these specific combinations of polymorphisms affect circadian phenotypes. The only genotype that differed between the two relevant combinations was the *Bmal1* genotype. This polymorphism is localized in the promoter region that includes a RORα transcription factor binding site, where the REV-ERB protein binds (Preitner *et al.*, 2002) to repress *Bmal1* expression. Therefore one can hypothesize that mRNA availability or the transcription rate of the *Bmal1* gene may be affected by the polymorphism. This differential expression would affect the amount of BMAL1 protein, consequently changing its rate of dimerization with the CLOCK protein, thus leading to different phases and/or amplitudes of expression to the other clock genes that could affect the regulatory loop timing. In studies on humans, differences in *Bmal1* phase of expression have been observed in blood cells (Teboul *et al.*, 2005), and animal studies have shown that *Bmal1* gene expression regulates other clock gene expression profiles, such as Per1 and 2 (Bunger *et al.*, 2002).

Viola *et al.* (2006) demonstrated strong effects of the *Per3* VNTR polymorphism in the sleeping and waking EEG structure and in the response to sleep loss. However, they mainly studied homozygotes and did not report upon the heterozygous phenotype for this polymorphism. In the context of the present results, it will be informative to test *Per3* VNTR heterozygotes under the same laboratory conditions but in combination with the *Bmal1* polymorphisms that we have described here.

The hypothesis of differential interactions between clock genes alleles associated with morningness-eveningness tendencies can be reasonably tested in our experimental set, which consisted of young adults selected for extreme diurnal preferences who were tested for the presence of specific combinations of clock gene polymorphisms. In this simple experimental setting it was possible to detect combined, but not individual polymorphism effects. It may be argued that our sample is small, which would decrease the power of the analyses, but we have chosen young subjects who are extreme morning or evening types, and selecting those individuals with extreme phenotypes for a quantitative trait, such as HO score, from a larger population, increases the power per individual genotyped (Ronin *et al.*,1998).

In spite of the logical rationale that the products of the clock genes interact to form the molecular clock machinery and that interactions of different alleles could therefore result in altered working of the machinery, very few studies have reported approaches to deal with this problem. In general, in animal studies, circadian behavior and circadian expression patterns of clock genes have been investigated in knockout mice with the absence of one or two components (double mutants) of the clock machinery. Data from these studies have shown that there is no circadian rhythmicity in the absence of more than one component (van der Horst *et al.*, 1999; Vitaterna *et al.*, 1999; Zheng *et al.*, 2001; Oster *et al.*, 2003). These data showed that the interaction between the clock components is necessary for molecular clock function, but failed to show how minor modifications, which still retain the function of the gene, may modify the regulation of circadian rhythms and the entrainment to environmental time cues. Thus, animal model studies (which are still lacking at the moment) that deal with naturally occurring polymorphisms would have a potential to clarify this question of clock gene interactions in regulating circadian behavior.

To our knowledge, this type of interaction has not previously been reported in humans, although several articles have reported associations between clock gene polymorphisms associated with circadian phenotypes related to the sleep/wake cycle (Katzenberg *et al.*, 1988; Ebisawa *et al.*, 2001; Archer *et al.*, 2003, Carpen *et al.*, 2005; Pereira *et al.*, 2005; Pedrazzoli *et al.*, 2007).

The present results demonstrate that interactions are accessible to study and should be expanded to search for haplotype interactions between clock genes, which should be more informative than single SNPs per gene. Taken together, the results show that it is possible to detect interactions between clock genes associated with human circadian phenotypes, thus pointing a way towards the construction of a clock gene polymorphism map applicable to human circadian biology.

## Figures and Tables

**Table 1:** Genotype combinations of polymorphisms in the *Per3*, *Per2*, *Clock* and *Bmal1* genes.

GC #	*Per3*	*Bmal1*	*Per2*	*Clock*	N	%
1	4/5	G/g	C/C	T/T	12	12.24
2	4/4	G/g	C/C	C/T	6	6.12
3	4/4	A/g	C/C	C/T	10	9.8
4	4/4	G/g	C/C	C/C	2	2.04
5	4/4	A/A	C/C	T/T	1	1.02
6	4/4	G/g	C/C	T/T	5	5.10
7	4/4	A/g	C/C	T/T	10	10.20
8	4/5	A/A	G/C	C/T	1	1.02
9	4/5	A/g	C/C	T/T	10	10.20
10	4/5	A/g	C/C	C/T	6	6.12
11	4/4	A/g	C/C	C/C	1	1.02
12	4/4	A/g	G/C	T/T	1	1.02
13	4/5	G/g	C/C	C/T	6	6.12
14	4/4	A/A	C/C	C/C	1	1.02
15	4/5	A/A	C/C	C/T	3	3.06
16	4/5	A/A	C/C	T/T	1	1.02
17	4/5	A/g	G/C	C/T	1	1.02
18	5/5	A/g	G/C	T/T	1	1.02
19	4/4	G/g	G/g	T/T	1	1.02
20	4/4	A/g	G/C	C/T	3	3.06
21	4/4	G/g	G/C	T/T	1	1.02
22	4/4	A/A	C/C	C/T	2	2.04
23	4/4	G/g	G/C	C/T	2	2.04
24	5/5	A/g	C/C	T/T	3	3.06
25	5/5	A/g	C/C	C/C	1	1.02
26	4/4	A/A	G/C	T/T	1	1.02
27	4/5	G/g	G/C	C/T	1	1.02
28	5/5	G/g	C/C	T/T	1	1.02
29	4/5	G/g	C/C	C/C	1	1.02
30	5/5	G/g	C/C	C/T	2	2.04
31	4/5	G/g	G/C	T/T	1	1.02
Total					98	100.00

GC#, Genotype combination number.

## References

[Albrechtetal2001] Albrecht U., Zheng B., Larkin D., Sun Z.S., Lee C.C. (2001). mPer1 and mPer2 are essential for normal resetting of the circadian clock. J Biol Rhythms.

[Archeretal2003] Archer S.N., Robilliard D.L., Skene D.J., Smits M., Williams A., Arendt J., von Schantz M. (2003). A length polymorphism in the circadian clock gene Per3 is linked to delayed sleep phase syndrome and extreme diurnal preference. Sleep.

[Barrettetal2005] Barrett J.C., Fry B., Maller J., Daly M.J. (2005). Haploview: Analysis and visualization of LD and haplotype maps. Bioinformatics.

[Bungeretal2000] Bunger M.K., Wilsbacher L.D., Moran S.M., Clendenin C., Radcliffe L.A., Hogenesch J.B., Simon M.C., Takahashi J.S., Bradfield C.A. (2000). Mop3 is an essential component of the master circadian pacemaker in mammals. Cell.

[Carpenetal2005] Carpen J.D., Archer S.N., Skene D.J., Smits M., von Schantz M. (2005). A single-nucleotide polymorphism in the 5'-untranslated region of the hPER2 gene is associated with diurnal preference. J Sleep Res.

[Carpenetal2006] Carpen J.D., von Schantz M., Smits M., Skene D.J., Archer S.N. (2006). A silent polymorphism in the PER1 gene associates with extreme diurnal preference in humans. J Hum Genet.

[DardenteandCermakian2007] Dardente H., Cermakian N. (2007). Molecular circadian rhythms in central and peripheral clocks in mammals. Chronobiol Int.

[Ebisawaetal2001] Ebisawa T., Uchiyama M., Kajimura N., Mishima K., Kamei Y., Katoh M., Watanabe T., Sekimoto M., Shibui K., Kim K. (2001). Association of structural polymorphisms in the human period3 gene with delayed sleep phase syndrome. EMBO Rep.

[ExcoffierandSchneider2005] Excoffier L.G.L., Schneider S. (2005). Arlequin v. 3.0: An integrated software package for population genetics data analysis. Evol Bioinform Online.

[HorneandOstberg1976] Horne J.A., Ostberg O. (1976). A self-assessment questionnaire to determine morningness-eveningness in human circadian rhythms. Int J Chronobiol.

[Iwaseetal2002] Iwase T., Kajimura N., Uchiyama M., Ebisawa T., Yoshimura K., Kamei Y., Shibui K., Kim K., Kudo Y., Katoh M. (2002). Mutation screening of the human Clock gene in circadian rhythm sleep disorders. Psychiatry Res.

[Katzenbergetal1998] Katzenberg D., Young T., Finn L., Lin L., King D.P., Takahashi J.S., Mignot E.A. (1998). CLOCK polymorphism associated with human diurnal preference. Sleep.

[KoandTakahashi2006] Ko C.H., Takahashi J.S. (2006). Molecular components of the mammalian circadian clock. Hum Mol Genet.

[Lowreyetal2000] Lowrey P.L., Shimomura K., Antoch M.P., Yamazaki S., Zemenides P.D., Ralph M.R., Menaker M., Takahashi J.S. (2000). Positional synthetic cloning and functional characterization of the mammalian circadian mutation tau. Science.

[Milleretal1988] Miller A.S., Dykes D.D., Polesky H.F. (1988). A simple salting out procedure for extracting DNA from human nucleated cells. Nucleic Acids Res.

[Mishimaetal2005] Mishima K., Tozawa T., Satoh K., Saitoh H., Mishima Y. (2005). The 3111T/C polymorphism of hClock is associated with evening preference and delayed sleep timing in a Japanese population sample. Am J Med Genet B Neuropsychiatr Genet.

[Osteretal2003] Oster H., van der Horst G.T., Albrecht U. (2003). Daily variation of clock output gene activation in behaviorally arrhythmic mPer/mCry triple mutant mice. Chronobiol Int.

[Pedrazzolietal2007] Pedrazzoli M., Louzada F.M., Pereira D.S., Benedito-Silva A.A., Lopez A.R., Martynhak B.J., Korczak A.L., Koike B.V., Barbosa A.A., et al (2007). Clock polymorphisms and circadian rhythms phenotypes in a sample of the Brazilian population. Chronobiol Int.

[Pereiraetal2005] Pereira D.S., Tufik S., Louzada F.M., Benedito-Silva A.A., Lopez A.R., Lemos N.A., Korczak A.L., D'Almeida V., Pedrazzoli M. (2005). Association of the length polymorphism in the human Per3 gene with the delayed sleep-phase syndrome: Does latitude have an influence upon it?. Sleep.

[Preitneretal2002] Preitner N., Damiola F., Lopez-Molina L., Zakany J., Duboule D., Albrecht U., Schibler U. (2002). The orphan nuclear receptor REV-ERB alpha controls circadian transcription within the positive limb of the mammalian circadian oscillator. Cell.

[RalphandMenaker1988] Ralph M.R., Menaker M. (1988). A mutation of the ircadian system in golden hamsters. Science.

[Robilliardetal2002] Robilliard D.L., Archer S.N., Arendt J., Lockley S.W., Hack L.M., English J., Leger D., Smits M.G., Williams A., Skene D.J. (2002). The 3111 Clock gene polymorphism is not associated with sleep and circadian rhythmicity in phenotypically characterized human subjects. J Sleep Res.

[Roninetal1998] Ronin Y.I., Korol A.B., Weller J.I. (1998). Selective genotyping to detect quantitative trait loci affecting multiple traits: Interval mapping analysis. Theor Appl Genet.

[Satohetal2003] Satoh K., Mishima K., Inoue Y., Ebisawa T., Shimizu T. (2003). Two pedigrees of familial advanced sleep phase syndrome in Japan. Sleep.

[Shearmanetal2000] Shearman L.P., Jin X., Lee C., Reppert S.M., Weaver D.R. (2000). Targeted disruption of the mPer3 gene: Subtle effects on circadian clock function. Mol Cell Biol.

[StoreyandTibshirani2003] Storey J.D., Tibshirani R. (2003). Statistical significancee for genomewide studies. Proc Natl Acad Sci USA.

[TaheriandMignot2002] Taheri S., Mignot E. (2002). The genetics of sleep disorders. Lancet Neurol.

[Tebouletal2005] Teboul M., Barrat-Petit M.A., Li X.M., Claustrat B., Formento J.L., Delaunay F., Lévi F., Milano G. (2005). Atypical patterns of circadian clock gene expression in human peripheral blood mononuclear cells. J Mol Med.

[Tohetal2001] Toh K.L., Jones C.R., He Y., Eide E.J., Hinz W.A., Virshup D.M., Ptácek LJ nd Fu Y.H. (2001). An hPer2 phosphorylation site mutation in familial advanced sleep phase syndrome. Science.

[vanderHorstetal1999] van der Horst G.T.J., Muijtjens M., Kobayashi K., Takano R., Kanno S., Takao M., Wit J., Verkerk A., Eker A.P.M., Leenen D. (1999). Mammalian Cry1 and Cry2 are essential for maintenance of circadian rhythms. Nature.

[Violaetal2006] Viola A.U., Archer S.N., James L.M., Groeger J.A., Lo J.C., Skene D.J., von Schantz M., Dijk D.J. (2006). PER3 polymorphism predicts sleep structure and waking performance. Curr Biol.

[Vitaternaetal1994] Vitaterna M.H., King D.P., Chang A.M., Kornhauser J.M., Lowrey P.L., McDonald J.D., Dove W.F., Pinto L.H., Turek F.W., Takahashi J.S. (1994). Mutagenesis and mapping of a mouse gene, Clock, essential for circadian behavior. Science.

[Vitaternaetal1999] Vitaterna M.H., Selby C.P., Todo T., Niwa H., Thompson C., Fruechte E.M., Hitomi K., Thresher R.J., Ishikawa T., Miyazaki J. (1999). Differential regulation of mammalian period genes and circadian rhythmicity by cryptochromes 1 and 2. Proc Natl Acad Sci USA.

[Zhengetal1999] Zheng B., Larkin D.W., Albrecht U., Sun Z.S., Sage M., Eichele G., Lee C.C., Bradley A. (1999). The *mPer2* gene encodes a functional component of the mammalian circadian clock. Nature.

[Zhengetal2001] Zheng B., Albrecht U., Kaasik K., Sage M., Lu W., Vaishnav S., Li Q., Sun Z.S., Eichele G., Bradley A. (2001). Nonredundant roles of the mPer1 and mPer2 genes in the mammalian circadian clock. Cell.

